# Long-Term Assessment of Baseline Blood Biochemistry Parameters in Rainbow Trout (*Oncorhynchus mykiss*) Maintained under Controlled Conditions

**DOI:** 10.3390/ani10091466

**Published:** 2020-08-20

**Authors:** Paolo Pastorino, Stefania Bergagna, Daniela Dezzutto, Raffaella Barbero, Marzia Righetti, Giulia Pagliasso, Laura Gasco, Maria Silvia Gennero, Elisabetta Pizzul, Alessandro Dondo, Marino Prearo

**Affiliations:** 1 The Veterinary Medical Research Institute for Piemonte, Liguria and Valle d’Aosta, via Bologna 148, 10154 Torino, Italy; stefania.bergagna@izsto.it (S.B.); marzia.righetti@gmail.com (M.R.); giulia.pagliasso@izsto.it (G.P.); mariasilvia.gennero@izsto.it (M.S.G.); alessandro.dondo@izsto.it (A.D.); marino.prearo@izsto.it (M.P.); 2 ASL TO4, Servizio Veterinario—Igiene degli allevamenti e delle produzioni zootecniche, via Aldisio 2, 10015 Ivrea, Torino, Italy; ddezzutto@aslto4.piemonte.it; 3 ASL TO4, Servizio Veterinario—Igiene degli allevamenti e delle produzioni zootecniche, via Regio Parco 64, 10036 Settimo Torinese, Torino, Italy; rbarbero@aslto4.piemonte.it; 4 Department of Agricultural, Forest, and Food Sciences, University of Turin, Largo P. Braccini 2, 10095 Grugliasco, Torino, Italy; laura.gasco@unito.it; 5 Department of Life Sciences, University of Trieste, via Giorgieri 10, 34127 Trieste, Italy; pizzul@units.it

**Keywords:** blood parameters, fish health, serum biochemistry

## Abstract

**Simple Summary:**

Baseline blood biochemistry parameter values, which are well established for humans and other higher vertebrates, are fundamental for diagnosis. In this study, provided for the first time are baseline biochemical values for rainbow trout maintained under controlled conditions (the density, water temperature and photoperiod were kept constant over time). Fish weight was found to influence the parameters analyzed. These findings permit the advancement of knowledge of blood parameters in fish and provide useful measures for assessing health status.

**Abstract:**

Twelve biochemical parameters were measured in serum blood samples from rainbow trout (*Oncorhynchus mykiss*) maintained under controlled conditions. Forty-five samples were taken every 3 months (T1–T4) over the course of one year to define baseline values. The effect of fish total weight (TW) was also evaluated. Principal component analysis showed a clear separation between T4 and T1, and T2 and T3, indicating an increase in certain biochemical parameters with weight. Linear regression analysis showed how TW significantly explained 11–67% of the variability observed for nine parameters out of 12. Pearson’s correlation matrix showed a significant positive correlation (*p* < 0.05) between TW and albumin, alkaline phosphatase, alanine-aminotransferase, creatinine, gamma-glutamyl transferase, magnesium, phosphorus and total protein. Furthermore, significant correlations (*p* < 0.05) between the majority of the biochemical parameters were found, indicating that growth can influence several parameters at the same time. The present study provides several useful baseline values for assessing the health of *O. mykiss*, indicating that fish weight is an important factor for interpreting the blood biochemical profile.

## 1. Introduction

Wild and reared fish are affected by intrinsic (e.g., age, sexual maturity and physiological condition) and external factors (e.g., hydrochemistry, temperature, handling and photoperiod) that are reflected in blood parameters [1,2]. Habitat and environmental conditions are also closely related to blood biochemical parameters [3]. Additionally, the stocking density, feeding ratio and feed composition can influence certain biochemical parameters [4].

It is difficult to determine whether measured parameters fall within the normal range for a given species. The determination of baseline blood biochemical parameters holds great importance for fish biology and pathology [5]. Published baseline values are available for wild and farmed fish from marine and freshwater environments; however, studies on farmed *Oncorhynchus mykiss*, one of the most economically valuable species on the Italian fish market [6], are scant to date [7,8,9]. Establishing baseline values for fish sampled directly in reared conditions is problematic due to the multiplicity of variables (especially environmental factors) that affect blood chemistry. Rearing conditions differ widely from farm to farm, even between those rearing the same species [7]. There is a need for determining baseline values for fish reared in controlled conditions, where environmental variables (e.g., temperature, hydrochemistry and density) are kept fairly constant during a study period. Moreover, information on the blood chemistry of fish reared under controlled conditions is lacking. To fill this gap, the aim of the present study was to measure twelve biochemical blood parameters to define baseline values in the serum of *O. mykiss* maintained under controlled conditions over the course of one year, and to evaluate the effect of weight on blood-based parameters.

## 2. Materials and Methods

### 2.1. Experimental Fish

This one-year study was carried out on 252 sex-reversed females of rainbow trout exhibiting a sterile filiform gonad [10] purchased from a private fish farm in northwest Italy. Thirty individuals were randomly selected for anatomopathological, parasitological, bacteriological and virologic examination following methods previously reported [11,12] to ensure that the fish were in optimal health condition. Fish were conditioned for 20 days before the beginning of the experiment. Histological changes to define liver steatosis were evaluated, and a semiquantitative severity score was assigned as reported previously [13]. The remaining fish (*n* = 222) were weighed at the beginning of the study (initial weight, 290.30 ± 26.21 g) and then randomly introduced (74 fish per tank) into three indoor fiberglass tanks with an overall cubature of 3 m^3^. The tank biomass was kept constant at 20 kg m^−3^ by raising or lowering the water level in each tank, based on fish growth and the fish biomass removed for blood chemistry analysis. In order to do that, fish were weighed once a month. Artesian well water (13 ± 1 °C) was supplied, with a water inflow rate of 8 L min^−1^. Physicochemical parameters were monitored daily with sensor devices supplied by Hanna Instruments Inc., Woonsocket, RI, USA: the water temperature (°C), dissolved oxygen (mg L^−1^) (HI 9147 oximeter), conductivity (uS cm^−1^) (HI 9033 conductivity meter), pH (HI 8125 pH/ORP meter), concentration of NH_3_/NH_4_^+^ (mg L^−1^), NO_3_^−^ (mg L^−1^) and PO_4_^3−^ (mg L^−1^) (multi-parameter benchtop photometer HI 86 83200-02). Feed (Optiline, Skretting, Mozzecane, Verona, Italy) was distributed by hand twice a day, every day. The daily feed quantity was set at 1.2% of tank biomass. The photoperiod was kept constant (12 h light/12 h dark). Mortality was checked every day.

### 2.2. Fish Sampling

Forty-five fish (15 from each tank) were sampled every 3 months (T1, T2, T3 and T4) for 1 year. The fish were immediately suppressed using an overdose (170 mg kg^−1^) of tricaine methanesulfonate MS-222 (Sigma-Aldrich, Milano, Italy) and weighed. Blood samples were collected in the morning (8–11 a.m.) by caudal vein puncture with a 5 mL syringe and transferred into Vacuette^®^ tubes containing serum clot activator in a 16 × 100 red cap-black ring (Greiner Bio-One GmbH, Kremsmünster, Austria). After blood collection, each fish was necropsied, immediately followed by bacteriological and parasitological examination, because disease status is known to influence blood parameters [1].

### 2.3. Sample Preparation and Analysis

For blood chemistry analysis, serum was obtained by centrifugation (15 min at 2000 rpm and 10 °C), visually inspected to rule out hemolysis that could influence the results, and stored at −80 °C until further analysis. The biochemical parameters considered were total proteins (PRTOT), albumin (ALB), alkaline phosphatase (ALP), alanine-aminotransferase (ALT), aspartate-aminotransferase (AST), cholesterol (CHOL), creatinine (CREAT), gamma-glutamyl transferase (GGT), magnesium (MG), phosphorus (PHOS), triglycerides (TRIGL) and urea (UREA). Serum concentrations were analyzed with an automated system photometer (I-Lab Aries Chemical Analyzer—Instrumentation Laboratory, Bedford, MA, USA).

### 2.4. Ethical Statement

The experimental protocol was designed according to the guidelines of the European Union Council 2010/63/EU for the use and care of experimental animals. The study was authorized by the Italian Ministry of Health (GR-2013-02355796).

### 2.5. Statistical Analysis

The Kolmogorov–Smirnov test was used to test the normality and homogeneity of variance for each biochemical parameter. Either one-way ANOVA or the Kruskal–Wallis test (when the assumption of normality was not stratified) was used to check for differences in biochemical measures and to compare the fish total weight at the four time points (T1, T2, T3 and T4). Tukey’s honest significant difference (HSD) test or Dunn’s multiple comparison test (after the Kruskal–Wallis test) was used as a post hoc test. Trends in biochemical parameters over the four sampling times were checked by principal component analysis (PCA). Pearson’s correlation matrix was used for correlation analysis between the biochemical parameters and total weight. Simple linear regression analysis was used to check the strength of the correlation (R^2^) between the biochemical parameters (dependent variables) and the total weight (independent variable). In this analysis, R^2^ describes the proportion or percentage of variance in the dependent variable explained by the variance in the independent variable. The criterion for significance was set at *p* < 0.05. Statistical analysis was performed using RStudio^®^ version 1.1.463.

## 3. Results

All fish intended for this study were healthy, since no alterations were observed at necropsy and the parasitological, bacteriological and virologic examinations were negative. The physicochemical parameters were within the recommended range values for rainbow trout farming [14] throughout the duration of the study. Significant weight gain occurred at the four time points (T1: 329.35 ± 38.38; T2: 450.03 ± 16.83; T3: 580.11 ± 33.93; T4: 735.35 ± 27.19 g; Kruskal–Wallis test, *p* < 0.001). No clinical signs or diseases were recorded during the study, except for mild hepatic steatosis (liver steatosis score = 1) at T4.

**Figure 1:**
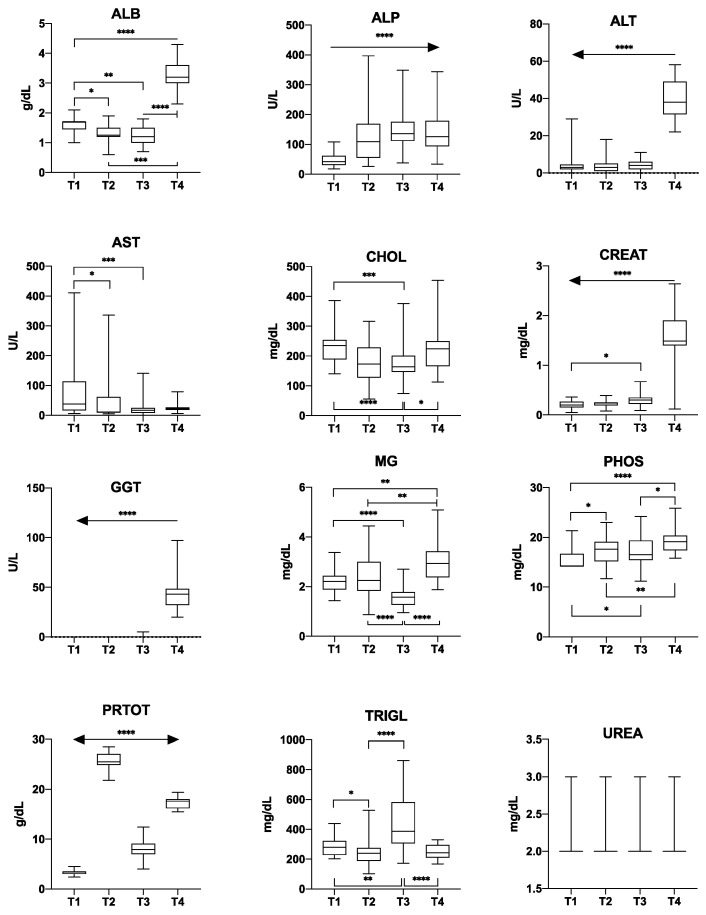
Boxplots of biochemical parameters measured in the serum of rainbow trout at four time points (T1–T4). Asterisks indicate significant differences according to Dunn’s test or Tukey’s test (* = *p* ≤ 0.05; ** = *p* ≤ 0.01; *** = *p* ≤ 0.001; **** = *p* ≤ 0.0001). ALB: albumin, ALP: alkaline phosphatase, ALT: alanine-aminotransferase, AST: aspartate-aminotransferase, CHOL: cholesterol, CREAT: creatinine, GGT: gamma-glutamyl transferase, MG: magnesium, PHOS: phosphorus, PRTOT: total proteins, TRIGL: triglycerides, UREA: urea.

The Kolmogorov–Smirnov test revealed that 10 out of 12 biochemical parameters (ALB, ALP, ALT, AST, CHOL, CREAT, GGT, PRTOT, TRIGL and UREA) showed non-normal distributions (*p* < 0.05), while MG and PHOS were normally distributed (*p* > 0.05). Figure 1 shows box plots with the concentration of each biochemical parameter at the four time points. The Kruskal–Wallis test highlighted a significant difference (*p* < 0.05) in concentration between the four time points for ALB, ALP, ALT, AST, CHOL, CREAT, GGT, PRTOT and TRIGL. Dunn’s multiple comparison test revealed significant differences between T1 and T2 for ALB, ALP, AST, CHOL, PRTOT and TRIGL (*p* < 0.05); between T1 and T3 for ALB, ALP, AST, CHOL, CREAT, PRTOT and TRIGL; between T1 and T4 for ALB, ALP, ALT, CREAT, GGT and PRTOT; between T2 and T3 for PRTOT and TRIGL; between T2 and T4 for ALB, ALT, CREAT, GGT and PRTOT; and between T3 and T4 for ALB, ALT, CHOL, CREAT, GGT, PRTOT and TRIGL. One-way ANOVA showed a significant difference (*p* < 0.05) in concentration between the four time points for MG and PHOS. Tukey’s HSD post hoc test highlighted significant differences between T1 and T2 for PHOS; between T1 and T3 for MG and PHOS; between T1 and T4 for MG and PHOS; between T2 and T3 for MG; between T2 and T4 for MG and PHOS; and between T3 and T4 for MG and PHOS.

**Figure 2:**
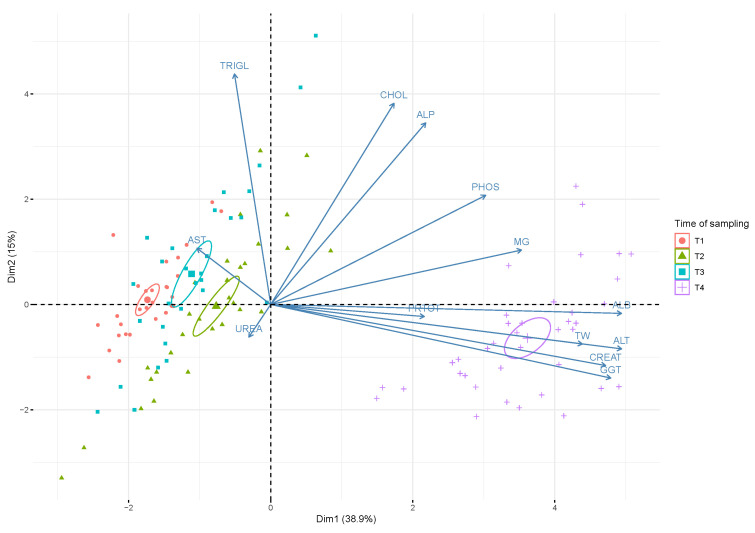
Biplot of scores and loadings from principal component analysis. The scores for each sampling time (T1, T2, T3 and T4) are denoted by a color and a symbol (largest symbol = average value). TW: total weight. Confidence ellipses (95%) are for the plotted values for each sampling time.

PCA (Figure 2) showed that the first (PC1) and the second (PC2) components accounted for meaningful amounts of the total variance (53.9%): PC1 explained 38.9% of the total variance and was positively correlated with ALB, ALT, CREAT; GGT, MG, PHOS and TW and negatively correlated with AST and UREA. PC2 explained 15% of the total variance and was positively correlated with ALP, CHOL and TRIGL and negatively correlated with UREA.

The four time points (T1, T2, T3 and T4) are arranged according to biochemical parameter value. There was a clear separation between T4 and T1, and T2 and T3. Blood samples taken at T4 are located on the right side of the plot in relation to the higher concentrations of ALB, ALT, CREAT, GGT, MG and PHOS; T1 and T2 are located on the left side of the plot in relation to the higher concentrations of AST.

Pearson’s correlation matrix showed a significant correlation between the majority of the biochemical parameters (Table S1). A significant positive correlation (*p* < 0.05) was found between TW and ALB, ALP, ALT, CREAT, GGT, MG, PHOS and PRTOT. A significant negative correlation (*p* < 0.0001) was found between TW and AST.

Regression analysis showed a significant linear relationship between TW and several biochemical parameters (ALB, ALP, ALT, AST, CREAT, GGT, MG, PHOS and PRTOT). TW significantly explained 42%, 15%, 56%, 11%, 57%, 55%, 57%, 67% and 11% of the variability observed for ALB, ALP, ALT, AST, CREAT, GGT, MG, PHOS and PRTOT, respectively.

## 4. Discussion

The monitoring of blood biochemical parameters is an integral part of non-invasive means of determining the health status of fish [15]. In this study, we provided, for the first-time, baseline biochemical values for rainbow trout, keeping fairly constant over time the density, water temperature and photoperiod. The distribution of many of the biochemical parameters was not normal, in line with previous observations [9,16]. Certain biochemical parameters (CHOL, MG, PHOS and TRIGL) we recorded are shared by previous studies [9,16]. On the contrary, other parameters (ALT, AST and GGT) of hepatic function showed different concentration values, depending on the time of sampling. In particular, an increase in ALT at T4 was observed, while AST levels decreased with fish growth. This trend may suggest hepatic injury, since there is a conspicuous increase in ALT compared to AST in most liver diseases such as steatosis [17]. Liver steatosis was observed at T4. Additionally, GGT values were significantly increased at T4. GGT activity is primarily observed in the biliary cells of the liver, and its increase in the blood may indicate liver impairment [18]. Particularly, the AST and ALT values at T1–T3 were much lower than those reported in the literature [9]. Additionally, creatinine showed significantly higher values at T4. However, since no increase in urea at that time and no kidney alterations were observed at necropsy, it can be anticipated that the increase in creatinine concentration was related to an increase in muscle activity [19]. On this path, the increase in blood ALP concentrations registered at T2 may result from increased osteoblastic activity during growth [20]. Variations in serum proteins and albumin have also been found in relation to growth [21]. This observation is consistent with our data.

In this study, we also focused on the effect of weight on biochemical parameters. Blood parameters are closely related to metabolic levels [22]; metabolic activity is higher during growth, with higher metabolic rates recorded for large than small fish [23]. This relationship is in line with our results, since the increase in fish weight (growth) paralleled the increase in several biochemical parameters, except for AST, which declined with increasing weight. Moreover, our findings showed that cholesterol and triglyceride levels were not significantly correlated with weight. This was probably due to diet and feeding, which remained constant for the duration of the study. The variation observed between sampling times was probably related to changes in hormone activity and growth, which influenced cholesterol and triglyceride levels [24]. Finally, our correlation matrix shows a positive correlation between most biochemical parameters, indicating that growth can influence several parameters at the same time.

## 5. Conclusions

In this study we provided for the first-time baseline biochemical values for rainbow trout maintained under controlled conditions. Moreover, we found that fish weight is an important factor that needs to be taken into account when interpreting the blood biochemical profile of rainbow trout to assess health status. Future areas of study are other factors that may influence blood chemistry and other species reared in aquaculture.

## Supplementary Materials

The following are available online at https://www.mdpi.com/2076-2615/10/9/1466/s1. Table S1: Pearson’s correlation matrix presenting biochemical parameters and total weight (TW) at each time point. Significance is highlighted in bold (*p* < 0.05).
