# Supplementary material for: Long-Term Assessment of Baseline Blood Biochemistry Parameters in Rainbow Trout (Oncorhynchus mykiss) Maintained under Controlled Conditions
Source: Animals (Basel). 2020 Aug 20;10(9):1466. doi: 10.3390/ani10091466 (PMC7552210; doi:10.3390/ani10091466)
Supplement: Supplementary file 1 [file animals-10-01466-s001.pdf]

Table S1. Pearson's correlation matrix presenting biochemical parameters and total weight (TW) at each time point. Significance is highlighted in bold ($p < 0.05$).

	ALB	ALP	ALT	AST	CHOL	CREAT	GGT	MG	PHOS	PRTOT	TRIGL	UREA	TW
ALB	**1**	**0.211**	**0.870**	-0.021	**0.442**	**0.774**	**0.855**	**0.576**	**0.407**	**0.176**	-0.144	-0.068	**0.639**
ALP	**0.211**	**1**	**0.180**	**-0.180**	**0.374**	0.131	**0.179**	**0.232**	**0.447**	**0.332**	**0.389**	0.009	**0.412**
ALT	**0.870**	**0.180**	**1**	-0.050	**0.285**	**0.838**	**0.871**	**0.456**	**0.336**	**0.193**	**-0.163**	0.027	**0.738**
AST	-0.021	**-0.180**	-0.050	**1**	**0.185**	**-0.171**	**-0.169**	0.002	-0.120	-0.154	0.143	-0.092	**-0.356**
CHOL	**0.442**	**0.374**	**0.285**	**0.185**	**1**	**0.211**	**0.163**	**0.520**	**0.268**	-0.015	**0.389**	-0.048	0.019
CREAT	**0.774**	0.131	**0.838**	**-0.171**	**0.211**	**1**	**0.799**	**0.391**	**0.237**	**0.200**	**-0.191**	-0.072	**0.749**
GGT	**0.855**	**0.179**	**0.871**	**-0.169**	**0.163**	**0.799**	**1**	**0.406**	**0.319**	**0.202**	**-0.242**	0.034	**0.744**
MG	**0.576**	**0.232**	**0.456**	0.002	**0.520**	**0.391**	**0.406**	**1**	**0.409**	**0.326**	**-0.179**	-0.034	**0.162**
PHOS	**0.407**	**0.447**	**0.336**	-0.120	**0.268**	**0.237**	**0.319**	**0.409**	**1**	**0.294**	**0.163**	-0.088	**0.369**
PRTOT	**0.176**	**0.332**	**0.193**	-0.154	-0.015	**0.200**	**0.202**	**0.326**	**0.294**	**1**	**-0.271**	-0.095	**0.272**
TRIGL	-0.144	**0.389**	**-0.163**	0.143	**0.389**	**-0.191**	**-0.242**	**-0.179**	**0.163**	**-0.271**	**1**	-0.021	-0.005
UREA	-0.068	0.009	0.027	-0.092	-0.048	-0.072	0.034	-0.034	-0.088	-0.095	-0.021	**1**	0.070
TW	**0.639**	**0.412**	**0.738**	**-0.356**	0.019	**0.749**	**0.744**	**0.162**	**0.369**	**0.272**	-0.005	0.070	**1**
